# Using deep learning to predict human decisions and using cognitive models to explain deep learning models

**DOI:** 10.1038/s41598-022-08863-0

**Published:** 2022-03-18

**Authors:** Matan Fintz, Margarita Osadchy, Uri Hertz

**Affiliations:** 1 Department of Computer Science, University of Haifa, Haifa, Israel; 2 Department of Cognitive Sciences, University of Haifa, 3498838 Haifa, Israel

**Keywords:** Computational models, Data processing, Machine learning, Human behaviour

## Abstract

Deep neural networks (DNNs) models have the potential to provide new insights in the study of cognitive processes, such as human decision making, due to their high capacity and data-driven design. While these models may be able to go beyond theory-driven models in predicting human behaviour, their opaque nature limits their ability to explain how an operation is carried out, undermining their usefulness as a scientific tool. Here we suggest the use of a DNN model as an exploratory tool to identify predictable and consistent human behaviour, and using explicit, theory-driven models, to characterise the high-capacity model. To demonstrate our approach, we trained an exploratory DNN model to predict human decisions in a four-armed bandit task. We found that this model was more accurate than two explicit models, a reward-oriented model geared towards choosing the most rewarding option, and a reward-oblivious model that was trained to predict human decisions without information about rewards. Using experimental simulations, we were able to characterise the exploratory model using the explicit models. We found that the exploratory model converged with the reward-oriented model’s predictions when one option was clearly better than the others, but that it predicted pattern-based explorations akin to the reward-oblivious model’s predictions. These results suggest that predictable decision patterns that are not solely reward-oriented may contribute to human decisions. Importantly, we demonstrate how theory-driven cognitive models can be used to characterise the operation of DNNs, making DNNs a useful explanatory tool in scientific investigation.

## Introduction

With the tremendous success of deep networks in image and language applications, this field has become a focus of attention in many other areas, including science. Deep networks have shown remarkable success in performing a variety of tasks with human-like and even super-human accuracy, leading to outperforming humans in some tasks^1–3^. However, in many scientific questions we are more interested in modelling and analysing data and strive for *explanations* rather than performing *predictions*^4–6^. In contrast to prediction tasks, it is not self-obvious how deep networks can help understand a natural process such as a cognitive task performed by humans (e.g., decision making). Here we propose a methodology for using a deep learning model to analyse a cognitive decision making process. The same scheme can be applied to other scientific problems.

One way of modelling a given process is by fitting a machine learning model to the data it produces. Ideally, we would like the model to be flexible enough to capture all predictable patterns. At the same time, we want it to be interpretable so that we can learn about the process by analysing the model. Unfortunately, these two goals are contradictory. Models with a high capacity, e.g., deep networks, are very difficult to understand and are generally considered black boxes^4,7,8^. On the other hand, models that are easily interpretable, e.g., models in which parameters can be interpreted as feature weights (such as regression) or models that maximize a simple rule, for example reward-driven models (such as q-learning) lack the capacity to model a relatively complex process. For example, since such models make specific assumptions about human behaviour and motivations, they may fall short if people’s behaviour is carried out in a different manner^9,10^.

A number of studies have used high-capacity deep-network models to understand a given cognitive process. The black-box nature of these models means that one cannot use model evaluation practices used in explicit, theory-driven models, such as parameter and model recovery^9,11^, at least not in a straightforward manner. For example, trying to recover the parameters by fitting a model to data generated by a specific implementation of the model will result in another uninterpretable black box. This calls for new approaches to model evaluation and characterisation, and previous works have used different methods to achieve interpretability. For example, one approach was to train many different models with different goals, and examine how they perform in predicting human behaviour, thus controlling for the model’s goal^12^, and another approach was to use adversarial examples that meant misleading a model and thus gaining insights on its operations^13^. These approaches take advantage of the domain knowledge on human motivations and performance in a task to explain the black-box model. An alternative approach, and the one used here, is to fit the deep network to human behaviour and examine how the model performs using experimental simulations. Such an approach was recently used to detect differences in decision making patterns between predefined groups of participants by their clinical conditions^14^. Similarly, we suggest utilising high-capacity deep-network models to capture complex cognitive processes in the general population, and then use explicit models developed to explain this cognitive process to characterise the black-box model.

Here we cast the problem of understanding human behaviour as an explainability problem in machine learning, and therefore use explicit and easy to interpret theory-based models to characterise the performance of the DNN model and the types of behaviour it captures. We demonstrate our approach of using both types of models, a high-capacity black-box model and explicit theory-driven models, to achieve both good fit and interpretability. We propose using a high-capacity deep-network model as an exploratory tool able to predict the outcome of the experiment as accurately as possible (up to noise levels in task performance) without relying on predefined theoretical models of the process. As mentioned above, the resulting model is a black box. The prediction of this “perfect” black box model can then be analysed using a set of explicit, theory-driven models. These models are designed to fit predefined and explicit patterns (simple models of the process) and are thus interpretable by design. While each such model alone cannot provide accurate predictions of the experimental data, these models can be used to characterise the black-box model. Once the black-box model is fitted to the experimental data, it can serve as a simulator of the process, generating new patterns of behaviour in novel, experimental settings. These model’s predictions can then be compared to the predictions of the explicit, theory-driven models in these novel settings to offer interpretation and gain scientific insight.

In this work, we exemplify the proposed approach to the problem of human decision making in a 4-arm restless bandit task^15^, where participants had to choose one of four options in order to obtain rewards, while the options’ reward magnitudes changed over time. Human behaviour in this task was previously modelled with a number of explicit, theory-driven models, such as reward-oriented learning and decision making (q-learning), choosing some preferred pattern either for periods of exploration or all the time (reward oblivious behaviour)^15–17^. On one hand, the chosen task is very simple (in the sense that the input is low dimensional), allowing us to train an accurate black-box model. On the other, the process that this task tries to capture is relatively complex in the sense that the dimension of the function operating on the low-dimensional inputs to generate output (i.e., cognitive processes) and variance in the outputs are high, requiring a high capacity model to fit the function.

## Results

### Task

**Figure 1:**
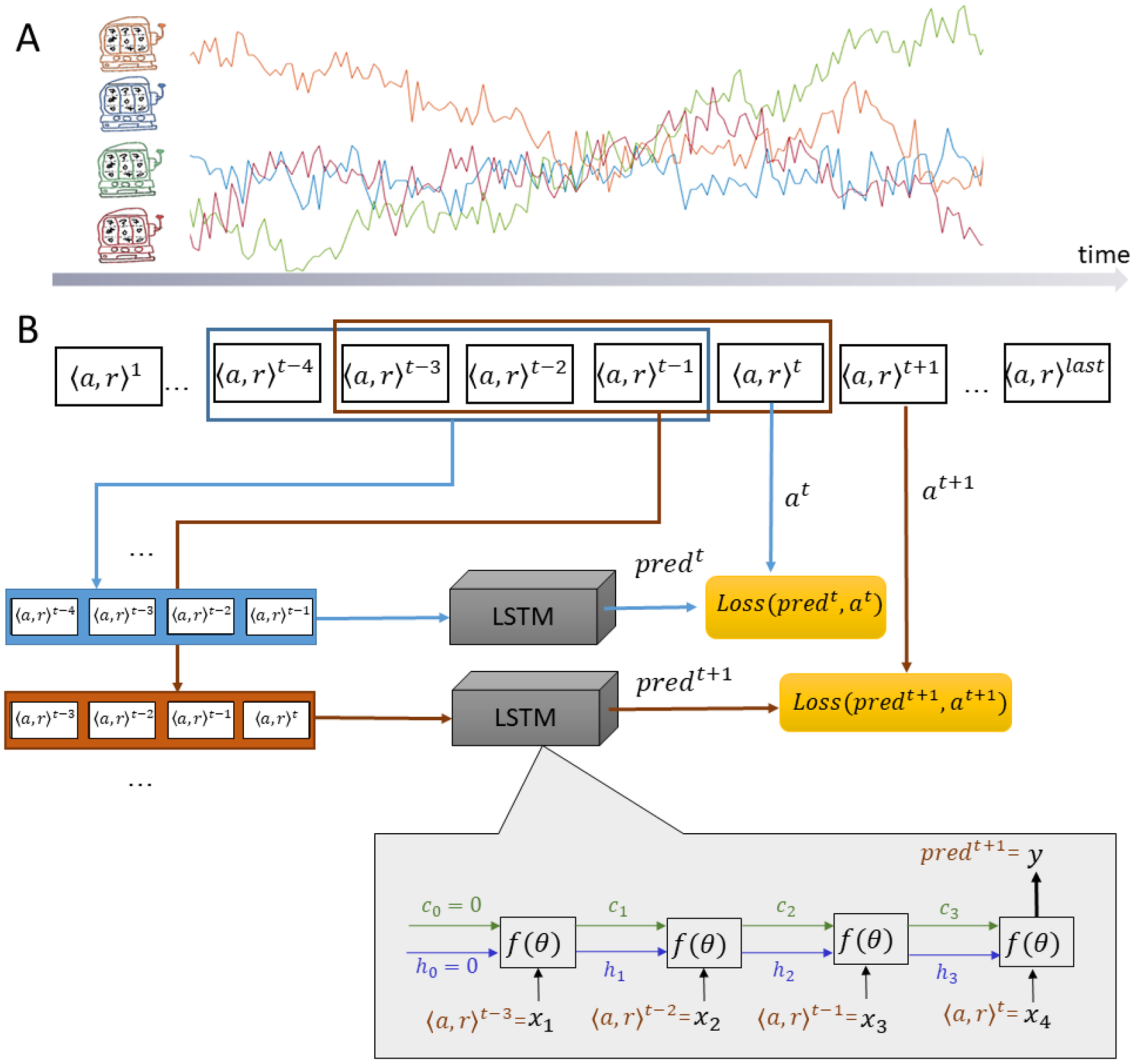
Experimental design and the exploratory LSTM model. (**A**) Experimental design—participants are asked to choose one of four options to gain rewards. The rewards associated with each option change slowly over time. (**B**) Sequences of four consecutive actions and rewards were used to train an LSTM model to predict the fifth action taken by the participant.

We examined a dataset of human decision making in a four-armed restless bandit task^18^. The experiment included 965 participants, who played 150 rounds of a four-armed bandit task online (Fig. 1). The probabilities of rewards drifted over 150 rounds. The participants were instructed to choose between four doors by clicking the door’s number to maximise the overall reward. We refer to these decisions as actions ${a}_{t}$*(*or *action at time t)*. Behind each door was a reward that drifted through the rounds in values ranging from 1 to 98, denoted ${r}_{t}$ *(*or *reward at time t)*. The rewards behind the four doors were predefined, and three such payoff structures were used, in line with previous work^15^.

### Exploratory DNN model: LSTM

We used Long Short Term Memory (LSTM) as an exploratory, high capacity, black-box model to predict human decision making in the above task. LSTM is a type of recurrent neural networks that allows modelling temporal dynamic behaviour by incorporating feedback connections in their architecture (Fig. 1). The choice of LSTM was mainly motivated by the sequential nature of the data. We did not explore models with larger capacity due to relative simplicity of the task. While it is possible to feed in the entire history of choices and rewards obtained for each participant to LSTM, there are two reasons to avoid this approach. First, this approach will result with different number of previous observations for each trial, predictions for early trials will be based on few observations, while predictions for late trials will be based on > 100 observations, which may bias the model’s prediction accuracy. Second, the restless nature of the task, where reward probabilities drift over time, make relying on infinite past history redundant. In Q-learning models, often used to model behaviour in such a task, the contribution of past experience is exponentially diminished (based on the learning rate). These reasons favour an approach that uses fixed length sequences of actions and outcomes to predict the next action. To this end, we trained the LSTM model to predict the participant’s action at time $t$, given his/her $K$ previous actions and the corresponding rewards (in times $t-K,..,t-1$). We chose $K=4$ as the number of action-reward steps to use in order to predict the next choice. Our experiments described in the Supplementary Materials show that while accuracy somewhat increased with greater lengths of sequences, the benefit of small increase in accuracy is outweighed by the gains in model simplicity, potential explainability (i.e. how easy it is to characterise the link between input and predictions), and generality (i.e., the ability to examine sequences from different time points, reward structure and participants using the same model).

The LSTM model was trained on short sequences of action-reward pairs extracted from the participants’ data (as shown in Fig. 1). The model can be viewed as a sequence of four units, corresponding to four consequent times. Each unit receives an input (denoted as ${x}_{i}, i=1,..,4$ in Fig. 1) comprising the participant’s action $a$ and reward $r$ from the previous step and the internal states (denoted ${h}_{i}$,${c}_{i}$), which carry on the information from the previous $1,\dots ,i$ steps. Each unit is a complex function parameterised by a large set of parameters, but these are shared by all units. The parameters are learned from the training set by minimising the disparity between the model’s prediction at the fifth step and the corresponding action of the participant. The disparity is formulated as a cross-entropy loss over four outcomes.

Our goal was to capture different behavioural types of participants in a single model. Due to the large capacity of LSTM, we believe that it can learn different policies from a data set including many participants and generalise over participants that were not in the training set. To this end we performed a fivefold cross validation in which the split was done over participants. Namely, each fold included 80% of the participants for training and 20% for testing. This way, instead of generalising the behaviour of the learned participant over time (as done in previous work^14^), we learn typical policies from one set of participants and generalise them over a new set of participants. The results of the cross-validation show that the trained LSTM model had an accuracy of 72.3%, indicating that it is capable of such generalisation.

### Explicit reward-oriented model

In order to examine how well the exploratory high-capacity model corresponded to a reward-oriented behaviour, i.e., actions that endeavour to maximise the acquired reward, we fitted a reinforcement learning model to the data using a q-learning algorithm^15^. This model assumes that participants make decisions based on the learned value associated with each option. The value of each option is updated whenever the participant chooses this option and obtains a reward according to the prediction error, i.e., the difference between the obtained reward and the options’ expected reward (current value). A free parameter, learning rate, controls the amount of updating in each trial and another free parameter, inverse temperature, controls the stochastic nature of choices (how likely are participants to choose a low value option). These parameters were estimated for the entire population, as was the case for the LSTM model, and were used to obtain model accuracy by comparing the model’s prediction of the participants’ choices.

This model is extensively used to model behaviour in such learning and decision making tasks^10,15,19,20^. Although many different extensions and elaborations of these simple mechanisms are used to capture different nuances in participants’ behaviour, they all share a common approach, which is that decisions are made to maximise reward, and that the history of obtained rewards drives the formation of reward expectations. These models are all reward-oriented in this sense. We choose to use a version of Q-learning which is relatively simple for the sake of easier interpretation, as we are interested in explainability, and for demonstration of our approach.

We found that the exploratory high-capacity model showed greater accuracy than the reward-oriented model in predicting participants’ actions in the task (Exploratory DNN Model accuracy: 72.3%, Reward-oriented accuracy: 68.2%).

### Explicit reward-oblivious model

We hypothesised that some participants may display behaviour that is not dependent on the action’s outcome, i.e., that is oblivious to reward^16,17,21^. During periods of uncertainty in outcomes, participant actions are less governed by reward-maximization, which corresponds to exploration^15^. Participants may use specific patterns of exploration, which are not reward dependent. For example, they may repeat sequences that were previously associated with outcomes^22,23^, follow a pattern of exploring one option at a time or simply follow a motor pattern^24^. Note that pattern based decision may coincide with reward-oriented behaviour, for example choosing the same action over and over when one option is much better than others^25^. Finally, the prevalence of reward-oblivious patterns may also be related to the lack of performance-based monetary incentives in the task.

To capture reward-oblivious patterns of behaviour, we build the Reward-Oblivious model. To train the model, we constructed a dataset from the original participants’ chains of actions, but without their rewards. These chains can be used to train different types of ML models. However, we used LSTM, because we wanted the differences between the exploratory LSTM and the reward-oblivious LSTM be directly attributed to the explicit presentation (or lack) of actions’ outcome and not to changes in the architecture and/or training procedure. Specifically, we trained Reward-Oblivious LSTM on the same data, but without the rewards, by chopping it into 4-step action sequences and trained the model to predict the following action (similar to the exploratory LSTM model). While actions, even stripped from rewards, may include information regarding the statistical regularities of rewards, it does not inform the reward-oblivious model about the reward itself. The model was fitted using short sequences of actions and it is guided by pattern completion. While the reward structure makes some patterns more prevalent (such as choosing the same option over and over), it does not mean that reward information affects other pattern completion predictions. Indeed, our experimental simulations (described below) examine explicitly those cases where reward-oriented and reward-oblivious models have contradicting predictions.

The results of fivefold cross-validation (with the same split as in exploratory LSTM) showed that the reward-oblivious LSTM produces less accurate predictions of human behaviour than the exploratory model (reward-oblivious accuracy: 69.9%). However, the reward-oblivious model is very good in pattern completion that is not reward-driven—it showed over 94% accuracy in predicting the next action for action sequences produced by our experimental simulations (detailed below). From these reasons, we believe that the reward-oblivious LSTM is able to capture action patterns that are not reward oriented.

### Comparison between the exploratory DNN model and the explicit models

To understand the predictions of the Exploratory model, we compared it with the explicit models across participants (averaged over time) and across time (averaged over participants) as detailed below.

**Figure 2:**
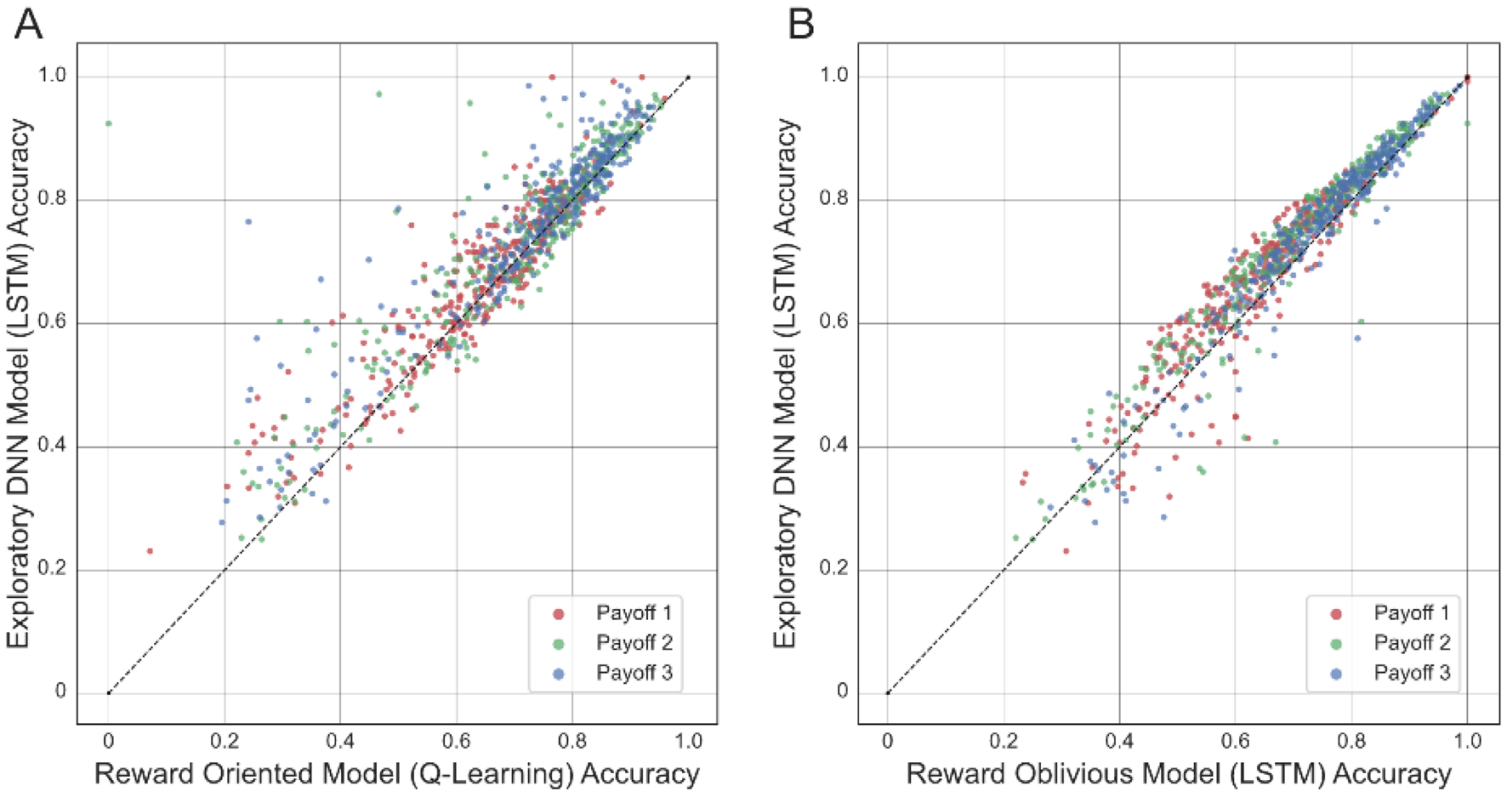
Comparison of the exploratory DNN and the reward-oriented (**A**) and reward oblivious (**B**) models’ accuracies across participants. Different colours mark the different payoff structure the participants experienced. Dots in the top triangle represent participants whose actions were more accurately predicted by the Exploratory DNN model than by the explicit models.

Figure 2 compares the accuracy of the exploratory DNN model and of the reward-oriented and reward-oblivious models across participants. While the advantage of the exploratory DNN model was not very big for some participants, for others this disparity was substantial. The analysis revealed a subset of participants whose actions were not captured by the reward-oriented model, suggesting that their choices were not affected much by their actions’ outcome, but were nevertheless captured and accurately predicted by the exploratory DNN model. In addition, the exploratory DNN model performed better than the reward-oblivious for most of the participants, with some participants displaying behaviour that was better captured by the reward-oblivious model (Fig. 2B).

**Figure 3:**
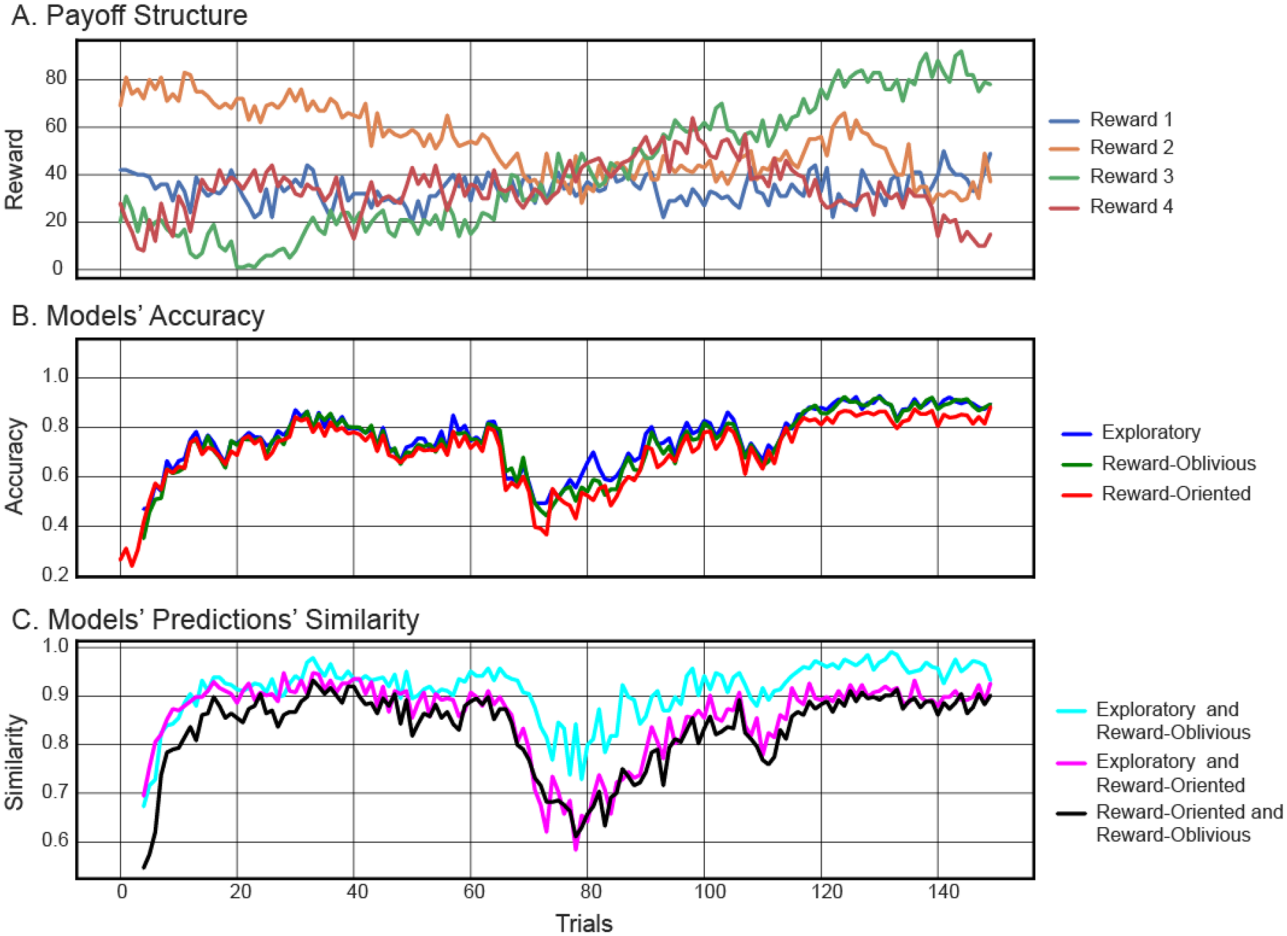
Analysis of models’ prediction over time–payoff structure 1. (**A**) Payoff structure indicates times when all options were similar and times when one option was distinctively better than others (payoff structure 1, for other payoff structure see Supplementary Materials). (**B**) Models’ prediction accuracy on trial-by-trial basis. Gaps between the lines indicate the differences in accuracies between the models. (**C**) Measure of similarity in predictions between pairs of models over time.

We compare the average accuracy of all three models in predicting human behaviour over time in Fig. 3. In this analysis it was possible to observe that the disparity between the exploratory DNN model and the reward-oriented model varied over time and was most apparent during the period of uncertainty in the reward structure (see the accuracy of the models with respect to the reward structure in Fig. 3 and in Supplementary Materials). Both models' accuracy levels were high when one option was markedly better than others. However, the exploratory model was more accurate when the options’ expected rewards were relatively close to each other. These times were associated with higher rates of exploratory choices, defined as times when participants choose options that are not associated with high reward^15^. The specific, trial-by-trial predictions made by the models is depicted in Supplementary Figs. SF8–SF10.

We observe that the reward-oblivious model in Fig. 3 is closer to the exploratory DNN model than the reward-oriented model in its accuracy. However, some discrepancy remains when there is no clear good option and the options’ outcomes are similar to each other. Predicting the next action for the constant pattern is easier than that of the exploration pattern (which is more diverse); this explains the better accuracy of the reward oblivious model when the best action is obvious (we believe that increasing the size of the training set could improve pattern prediction during uncertainty periods).

To better understand the relationship between the reward-oblivious and the reward-oriented models’ predictions, we examined the overlap in their predictions over time (Fig. 3C). At each time point we compared their accuracy pattern—whether they were accurate or not in predicting each participant’s actions. If both models predicted correctly, we marked this mutual success, expressed as 1 in a similarity vector. If both failed, it was also marked 1 in the similarity vector. A mismatch in predictions was marked 0. Summing this similarity vector over all participant in each reward structure in a specific time point gave an indicator of similarity in predictions between the two models.

We observed that in many cases both reward-oriented and reward-oblivious models agreed, especially when one option was constantly better than others and therefore selected repeatedly (Fig. 3C). From a reward maximisation point of view, such a repeated choices pattern is the hallmark of exploitation—choosing the known best option^15^. From a reward-oblivious point of view, this was a pattern that regularly appeared in the choices sequences and therefore was relatively easy to capture and predict.

However, similarity in predictions decreased when there was no single obvious good option. As shown before, these were times where participants explored the different choices and the overall accuracy of all models in predicting participants’ choices decreased. The fact that models’ similarity decreased as well indicates that the models made different predictions in these times, suggesting that outcome information made a difference. Importantly, these were also times when the gap between the reward-oriented and the exploratory DNN model was the greatest, suggesting that maybe the advantage of the exploratory DNN model came from incorporating non-reward oriented choice patterns, which were captured by the reward-oblivious model, in order to form its predictions. Even though both the reward-oblivious and the reward-oriented model are less accurate than the exploratory DNN model in predicting human behaviour during periods of considerable uncertainty, they capture different aspects of human behaviour that jointly constitute the policy learned by the exploratory DNN model. It is important to note that the exploratory DNN model was trained to predict one choice following a sequence of four action-reward pairs. As such, examining its performance over the entire task (150 trials) is helpful in detecting the relationship between predictions and task structure, but its ability to characterise and evaluate the model’s operation is limited and a different approach is needed.

### Experimental simulations

Examining the time course of models’ accuracy and similarity revealed that under some conditions the reward-oriented model and the reward-oblivious model gave very different predictions, and that both models may contribute to the performance of the exploratory DNN model. To better characterise these different predictions and their contribution we designed experimental simulations, where models’ predictions could be compared under specific action-reward sequences. The experiments were inspired by action-reward sequences that could occur in the data, such as increasing or decreasing rewards or a pattern of choices, but were not selected from the data itself and did not occur in the data. This was done in order to design novel settings to test our model. In all experimental simulations we introduced novel action-reward sequences in the models after these had been trained on participants’ actual behaviour. As we did not use predictions over entire population as before, we used the distance between the models’ predicted probabilities for all four actions in order to compare the model’s predictions. In this way if two models predicted similar probabilities to each option, for example all were close to 25%, they would be similar to each other.

We examined the predictions of the reward-oriented and reward-oblivious models for three action sequences coupled with three different reward sequences, resulting in nine overall action-reward sequences (Table 1). We used a constant action pattern, i.e., choosing the same option over 4 consecutive trials, an all-different pattern where each option is selected once (e.g., 1–2–3–4) and an alternating pattern where one alternates between two options (e.g., 1–2–1–2). The rewards coupled with these action sequences either stayed constant (e.g., 40–40–40–40), increased over time (e.g., 20–30–70–80) or decreased over time (e.g., 80–60–40–20).

**Table 1:** Comparison of the reward-oriented vs reward-oblivious models’ predictions on action-reward examples.

Action and reward pattern input to models					Models’ prediction of probabilities for each action		
Reward pattern	40	40	40	40	Reward-oriented	Reward-oblivious	Exploratory
Action patterns	1	1	1	1	0.18,0.27, 0.27, 0.27	0.78, 0.14, 0.05, 0.03	0.77, 0.12, 0.07, 0.04
	1	2	1	2	0.2, 0.2, 0.3, 0.3	0.5, 0.24, 0.18, 0.08	0.74, 0.07, 0.1 , 0.08
	1	2	3	4	0.25, 0.25, 0.25, 0.25	0.5, 0.3, 0.16, 0.04	0.52, 0.24, 0.15, 0.09
Reward pattern	20	30	70	80	Reward-oriented	Reward-oblivious	Exploratory
Action patterns	1	1	1	1	0.48, 0.17, 0.17, 0.17	0.78, 0.14, 0.05, 0.03	0.99, 0.004, 0.001, 0.005
	1	2	1	2	0.26, 0.38, 0.18, 0.18	0.5, 0.24, 0.18, 0.08	0.03, 0.9, 0.025, 0.05
	1	2	3	4	0.07, 0.1, 0.35, 0.48	0.5, 0.3, 0.16, 0.04	0.03 , 0.073, 0.077, 0.82
Reward pattern	80	60	40	20	Reward-oriented	Reward-oblivious	Exploratory
Action patterns	1	1	1	1	0.1, 0.3, 0.3, 0.3	0.78, 0.14, 0.05, 0.03	0.11, 0.32, 0.45, 0.12
	1	2	1	2	0.27, 0.13, 0.3, 0.3	0.5, 0.24, 0.18, 0.08	0.22, 0.22, 0.38, 0.18
	1	2	3	4	0.5, 0.27, 0.15, 0.08	0.5, 0.3, 0.16, 0.04	0.8 , 0.17, 0.01, 0.02

The predictions are represented as four probabilities for choices, one for each action. Reward-oriented model predicts the highest probability for the action that maximises the expected reward, while the reward-oblivious model predicts the highest probability for the action that completes the pattern that exists in the sequence of previous actions. Identifying the conditions under which the Exploratory model’s predictions converge with the reward-oriented model’s, and those in which it converges with the reward-oblivious model’s, can be used to characterise and explain the Exploratory model.

The reward-oblivious model predicted the highest probabilities to actions that completed or repeated a pattern, regardless of rewards (Table 1 for a concrete example, see Supplementary Files for full tables of simulations). It predicted that the participant would keep on choosing the same option in the constant pattern. In the all-different and alternating patterns, it predicted that the pattern would repeat itself, i.e., the first choice in the 4-action sequence would be chosen again. For example, after observing a pattern of 1–2–3–4 it predicted that the participant would choose 1. This model’s predictions were not dependent on the reward pattern, as this was not inputted to the model.

The reward-oriented model’s predictions were tightly linked to the rewards (Table 1). For example, when observing the action sequence 1–2–3–4 coupled with the reward sequence 20–30–70–80 it predicted highest probability to choosing option 4, as this option was associated with the highest reward, but when the same action pattern was coupled with decreased rewards, 80–60–40–20, it predicted the highest probability to choosing option 1, as it yielded the highest reward. Note that some predictions of the reward-oriented model converged with the predictions of the reward-oblivious model, as sometime the action pattern agreed with the reward pattern, just as in the descending-rewards and all-different action example.

As these examples demonstrated, some action-reward sequences led to different predictions by the reward-oblivious and reward-oriented models. In the full experimental simulations, we expanded these sequences to test a variety of action-reward sequences, falling into the same categories of action patterns (constant, alternating, all-different) and rewards (constant, increasing, decreasing) (see “Methods” section and Supplementary Materials for full details of the sequences). In this wide array of action and reward sequences we observed that the reward-oblivious model always predicted pattern completion, similar to the results shown in Table 1, indicating that this model indeed captures motor patterns of actions (see Supplementary Materials for detailed predictions by all models).

**Figure 4:**
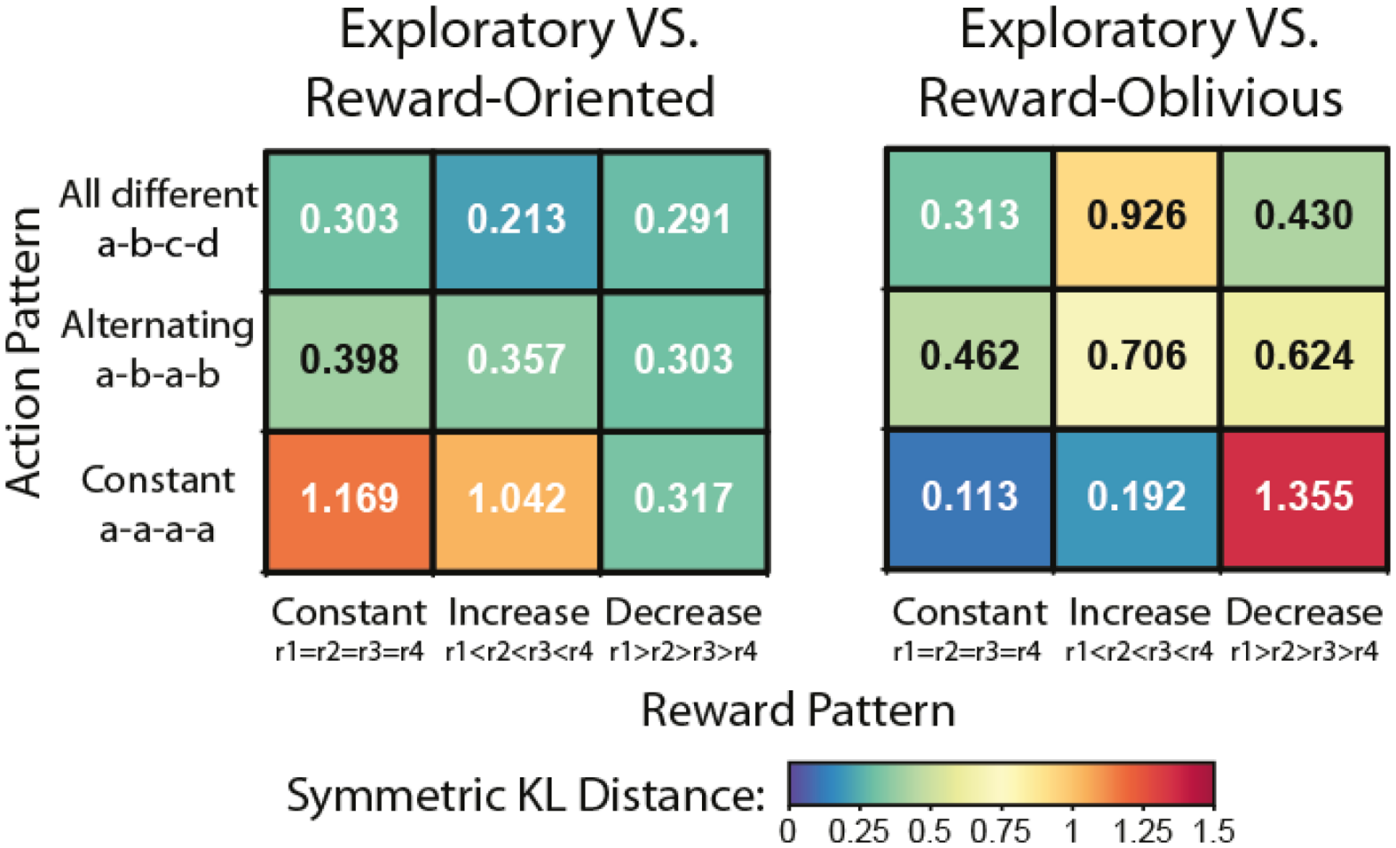
Comparison of models’ predictions in experimental simulations. We simulated the models with different sequences of actions and rewards, aimed at differentiating between the two explicit models’ predictions, and examined how these corresponded to the predictions made by simulating the exploratory DNN model. We clustered together sequences based on their action-pattern and reward-pattern categories in the different cells. Symmetric KL distance was measured between the model’s predictions; low value indicates small distance. Note that in some simulation conditions the exploratory DNN model’s predictions converged with the reward-oblivious model’s and not with the reward-oriented model’s predications, and vice versa (for example, in the constant action row).

Using the predictions from all three models, we then examined in which conditions the general model’s predictions were most similar to those of the reward-oblivious model, and under which conditions it was most similar to the reward-oblivious conditions (Table 1). As the predications of the explicit models under the experimental conditions were related to a explicit process, such a step can help characterising the predictions and operations of the exploratory model. We used a symmetric Kullback–Leibler (KL) distance to measure the distance between the predictions made by the models (see “Methods” section). In Fig. 4 we present the pairwise comparison of the exploratory model and the two explicit models’ predictions for the different combinations of action and reward sequences. These were clustered together to provide an easy overall description of the similarity in predictions between the three models.

A number of patterns emerged from these comparisons. In some cases, all models give similar predictions, in others the exploratory DNN model’s predictions seem to converge with the predictions made by the reward-oriented model, while in yet others they converge with the predictions of the reward-oblivious model. Examining the combinations of reward and action sequences that underlie these cases can help characterise the operation of the exploratory DNN model in terms of sensitivity to action patterns and association with rewards.

A differentiation between the exploratory model’s convergence with reward-oriented and reward-oblivious models was observed when the action pattern was constant (a–a–a–a) (bottom rows in Fig. 4, Table 1). When the constant action-pattern was accompanied by a constant reward pattern (r1 = r2 = r3 = r4), the exploratory model gave similar predictions as the reward-oblivious model, i.e., kept on choosing the same action. The model diverged from the reward-oriented model’s predictions in these situations, as these cells include conditions where the constant rewards were low (< 50) and high (> 50) and the reward-oriented model’s predictions were highly dependent on the value of rewards (see example in Table 1). However, when the reward pattern was a decreasing pattern (r1 > r2 > r3 > r4), the exploratory model converged with the reward-oriented predictions of switching action and differed from the pattern-completion prediction of the reward-oblivious model.

Another differentiation was observed when the reward pattern was an increasing pattern (r1 < r2 < r3 < r4) (middle columns in Fig. 4). When the increasing reward was accompanied by a constant action pattern, the exploratory model converged with the reward-oblivious pattern’s completion prediction and was less sensitive to the amount of the reward, i.e., predicted pattern completion even if the final reward observed was low (such as in the case of 10–20–30–40 points). However, when the increasing reward pattern was observed in four different actions, the exploratory model’s predictions converged with the reward-oriented predictions in predicting the choice of the option associated with the highest reward.

Finally, in some cases the exploratory model’s predictions were similar to both reward-oriented and reward-oblivious predictions. This is apparent when the action pattern includes four different actions and the reward pattern is a descending one (Fig. 4 top right cell, example in Table 1). In these cases, pattern completion and choosing the action with the highest reward both point to the same action—choosing the first action.

Our experimental simulations suggest that the exploratory model relies on the rewards associated with the different actions, most decidedly so when one option is clearly better than others. However, when the reward structure was less clear about a single best option, for example when the rewards were constant, the exploratory model tended to predict pattern completion actions in a way that was less sensitive of the specific rewards. This suggests that participants were mixing these strategies and relying more on action patterns when the rewards did not indicate a clear best action.

For the sake of simplicity of our demonstration, we focused here only on a few experimental conditions. Note that in some cases this relationship was not so straightforward and required a closer investigation of the prediction matrix, for example when the reward was constant and the action pattern included all actions (a–b–c–d). There, the exploratory model’s predictions were a mix of the reward-oblivious and reward-oriented model’s predictions—a tendency to give similar probabilities to all actions such as the rearward-oriented, with a slightly higher probability for the pattern completion action. Taken together, the results of the experimental simulations suggest that both action-pattern repetition and reward maximisation strategies were encoded by the exploratory DNN model and may contribute to the higher accuracy observed across time and participants.

## Discussion

In this paper we aimed to demonstrate how exploratory DNN model and explicit, theory-driven models could be used in tandem to gain novel scientific insights. We took advantage of DNN models’ great capacity and ability to capture regularities in data, and used them as exploratory tools for examining the scope of predictable human behaviour in a widely-used learning and decision making experimental paradigm. We observed that our exploratory DNN model gave more accurate predictions than an explicit reward-oriented reinforcement learning model. This disparity was more pronounced when the reward uncertainty was high. To characterise what made the exploratory DNN model more accurate, we trained another explicit model, the reward-oblivious model, that did not have direct access to the outcomes of actions in this task. The reward-oblivious model’s predictions were associated with action pattern completion—it was likely to predict actions that complete a sequence of actions. Using experimental simulations, we found that the exploratory DNN model converged with the reward-oriented model’s predictions under some experimental settings, and with the reward-oblivious model’s predictions in others. Specifically, the exploratory model predicted pattern completion when all actions were expected to lead to similar rewards, i.e., there was no clear best option to choose. It demonstrated that during high uncertainty, when the best course of action is not clear, people may explore options following a *predictable* pattern of action, which is not necessarily and immediately reward-oriented. This observation demonstrates the usefulness of DNN models as exploratory tools in cognitive science.

Our approach highlights the benefits of using a high-capacity exploratory model as a scientific investigation tool. Fitting such a model to our data can suggest how much of the data can be explained and predicted, detecting patterns of regularities beyond current theory driven investigations^26^. DNNs can therefore expand researchers’ field of view, casting light on behavioural patterns so far treated as noise by theory-driven approaches. In a recent work, Dezfouli et al.^14^ showed that a DNN can capture meaningful differences in behaviour between groups of participants diagnosed with bipolar and unipolar disorders, and healthy controls. Moreover, the DNN model was able to capture irregularities in the clinical populations’ choice patterns, that could be used to inform future research. Here we demonstrated that a DNN model could uncover patterns of behaviour made by healthy populations, that included both reward-oriented process and reward-oblivious, pattern completion. These two examples suggest that behavioural patterns in learning and decision making task include a number of different strategies, which are meaningful, and predictable. For example, in the learning and decision making paradigms like the one used here, divergence from reward-oriented behaviour was labelled as random explorations^15,27^, or as a stickiness heuristics, i.e. a tendency to repeat one’s choice^15^. Others identified pattern completion responses where participant repeat sequences that were useful before^23^, or follow a similar motor response^28^. It is therefore clear that gaining a better understanding of how much of the ‘noise’ that is not explained by reward-oriented or other explicit theory driven models is actually predictable, using a high capacity exploratory model, can greatly aid in directing future scientific and theory driven investigations.

However, after the first exploratory step is taken, a second explanatory step is needed. This problem is not unique to scientific exploration, but is encountered by many researchers and practitioners trying to provide explanations of how their black-box models operate in order to gain a better understanding of how they work and what they predict, and to promote trust in these algorithms^7,8^. This is referred to as the explainability problem. Different tools and approaches are being developed for this purpose, for example using visualisation to make linear regression models easy and quick to understand, and matching decision tree models to provide a systematic description of the model’s behaviour^29–32^. In cognitive neuroscience, another approach to this problem is to use behavioural experimental tools to explain the model’s behaviour^14,33^. One way to carry out this task is by examining the different experimental settings that make the model fail, known as adversarial examples^13^, which has a long tradition in cognitive psychology, from the use of visual illusions to study perception to the characterisation of biases in decision making^34^. Here we used cognitive models that provide explicit predictions, reward-oriented and reward-oblivious models, to characterise the performance of our exploratory DNN model. Using experimental simulations designed to differentiate between these two models, we were able to chart the gap between the general DNN model and the traditional reward-oriented approach. Explicit cognitive models can therefore be useful for explaining the operation of DNNs and for guiding future investigations following the exploratory use of DNN models.

A number of limitations should be considered for future implementation of our approach. First, using high capacity models also means using models with many free parameters, much more than in most explicit models. This means that there is a danger of overfitting the model, especially if the dataset to be predicted is small. However, our approach uses the DNN model to indicate what may be predicted, and then uses explicit models with few free parameters to characterise it, thus reducing the problem of overfitting by focusing on the predictions that could be characterised by explicit models. Another open question has to do with modelling individual participants. Our approach was aimed at predicting behaviour at the level of the entire population, using short action-reward sequences from all participants, and uncovered different strategies for behaviour. It was therefore able to predict the behaviour of individuals that did not follow a reward-oriented strategy. The general DNN could therefore potentially use only a small amount of a single-participant history of actions and rewards to predict her next choice. However, the use of DNN for individual level predictions is beyond the focus of this work and will be investigated in future research.

Taken together, our work demonstrated how DNN models can be used to uncover hitherto ignored human behaviours, and how explicit models can be used to characterise these black-box models. While we demonstrated our approach using a decision making task, we suggest that it is applicable in other scientific domains, either in psychology or in other scientific fields, where inference of underlying processes from noisy data is needed. In addition, we suggest that explicit theory-driven models could be used to characterise black-box models, especially using an experimental settings comparing these models. Such characterisation may be useful not only for promoting scientific knowledge, but also in communicating model’s performance to practitioners and the general public, for example by providing an explicit description of the way a black-box model behaves.

## Methods

### Task

We examined a dataset of human decision making in a four-armed bandit task collected by Bahrami and Navajas^18^. The dataset is available online, and the authors gave us an explicit permission to use the data. The original experiment was approved by their local Institutional review board, and all methods were performed in accordance with relevant guidelines and regulations. The experiment was carried out online, and included 965 participants playing 150 rounds of a four-armed bandit task. In this task participants had to choose one of four options in each trial, in order to obtain rewards (points in the game, no performance based monetary reward was given in this experiment) (Fig. 1). Note that the lack of monetary incentive may make participants’ responses noisier. However, the general good fit of reward-oriented model to participants’ behaviour indicates that they were oriented towards maximizing reward to some extent. We refer to these choices as ${a}_{t}$ (or *choice at time t*). The amount of rewards associated with each option was initially set to a value between 0 and 98 points, and drifted over time (standard deviation σ = 2.8, more details in the Supplementary Materials), so participants had to keep on tracking the outcomes to pick the highest paying option. Rewards are denoted by ${r}_{t}$ (*or reward at time t*). Participants faced one of three payoff structures generated in this manner (see Fig. 1, Supplementary Figs. SF3, SF4). Participants had to reach a decision within four seconds, and failing to do so moved them to the next trial with no reward. Of 965 participants in the dataset, only 127 players completed all 150 rounds, while others missed at least one trial. The average number of rounds per participant was ~ 145. The average reward in each round across all participants was 65.8. Behavioural measures, i.e. mean choices and choice variance, are reported in the Supplementary Materials.

### Data preparation for models

For the exploratory DNN model, we created a data set by splitting the original data of each participant into 4-step action-reward sequences ${<(a}_{t-4}, {r}_{t-1}), \left({a}_{t-3}, {r}_{t-3}\right), \left({a}_{t-2}, {r}_{t-2}\right), ({a}_{t-1}, {r}_{t-1})>$ using a sliding window (Fig. 1). After evaluating the gain in accuracy resulting from the addition of each step (see Fig. SF1 in the Supplementary Materials), we chose 4-step sequences as a trade-off between efficient use of the data and the memory needed for successful performance in the task. The model makes the prediction at time $t$ given the previous 4 steps, thus the first prediction is for the 5th round.

We excluded missed trials from the sequences, causing 4% of the sequences to include a gap (i.e., missed response), for instance a sequence where action 6 was missing can be ${<(a}_{4}, {r}_{4}), \left({a}_{5}, {r}_{5}\right), \left({a}_{7}, {r}_{7}\right), ({a}_{8}, {r}_{8})>$, with the model predicting action 9. Almost all of the sequences with a gap missed only a single round. Since the drift in rewards associated with the options was slow, we included sequences with gaps in the dataset. The order of the resulting sequences across all participants was shuffled (as we assume no continuity beyond the 4-step sequence).

The reward-oblivious model inputs a sequence of actions without the corresponding rewards ${ <(a}_{t-4}), \left({a}_{t-3}\right), \left({a}_{t-2}\right), ({a}_{t-1})>$. The sequences were produced using the same procedure as for the exploratory DNN model.

The reward-oriented model was provided the total action sequences of the participants (i.e., 150 actions and rewards), and was fitted for each participant independently.

### LSTM models

Long short term memory (LSTM) is a type of recurrent neural networks (RNN), which allows modelling temporal dynamic behaviour by incorporating feedback connections in their architecture^35^. Our exploratory and reward-oblivious models are both LSTM models with four units. We used a single layer LSTM, as the task itself is simple and there is no need for a deeper network. Each LSTM cell has 64 hidden units, trained for 300 epochs with a batch size of 2048. Both the hidden layer size and the number of epochs were determined after running a grid search for hyperparameter tuning, by fitting the model with different configurations of these parameters, using the fivefold cross validation model fitting. The last unit includes a 4-way softmax layer for outputting the probability of choosing each of the 4 doors. For the general model, the input ${x}^{<t>}$ to the unit $t$ comprises the previous action and previous reward $\left({a}_{t-1}, {r}_{t-1}\right)$, while the reward-oblivious model inputs only the previous action $({a}_{t-1})$.

The exploratory DNN and reward-oblivious models were trained on the participants’ data as explained above using the categorical cross-entropy loss:

1 $$Loss= - \frac{1}{N}\sum_{i=1}^{N}{y}_{i}\text{log}\widehat{{y}_{i}}.$$

The models were trained using Adam optimiser with a learning rate of 0.001, beta1 = 0.9 and beta2 = 0.99. We implemented the LSTM models in TensorFlow, under the Windows operating system, with the GTX 970 graphic card.

We evaluated the LSTM-based model using a fivefold cross validation, where we trained the model using data from 80% of the participants and tested its accuracy in predicting the choices of the hold out 20% of the participants. This process was done 5 times, with no overlap of the test group (i.e. model predictions were evaluated only once per participant). The evaluation phase on the hold-out set in each fold was performed without sliding window.

### Q-learning model

For the reward-oriented model we used a reinforcement learning model, q-learning^36,37^, which is commonly used to model the behaviour of human participants in similar tasks to the one used here^15^. The model assumes that decisions are driven by the expected reward of each option, and that these expected rewards are learned on a trial-by-trial basis by updating the learner’s expectations (known as q values) based on prediction errors, the difference between the obtained reward and the expected reward. In our case, in each trial the participant makes a decision which of the four doors to open, noted as action ${\text{a}}_{\text{t}}$, and receives a reward ${\text{r}}_{\text{t}}$. In each round the expected value of the chosen action, noted as ${Q}_{t}({a}_{t})$ is updated according to Eq. (2):

2 $${Q}_{t+1}\left({a}_{t}\right)={Q}_{t}\left({a}_{t}\right)+\alpha {\delta }_{t},$$

where $0\le {\alpha }\le 1$ is a free learning rate parameter and ${\delta }_{t}$ is the prediction error ${\delta }_{t}={r}_{t}-{Q}_{t}({a}_{t})$. Q-values were initiated with the value of 50, which was in the middle of the rewards range. Note that this initial value was overridden as evidence accumulated over time.

In each trial t, the model assumes that the participants make their choices according to a softmax distribution based on the q-values they learned so far:

3 $${p}_{t}\left(a\right)=\frac{{e}^{\beta {Q}_{t}\left(a\right)}}{{\sum }_{i}{e}^{\beta {Q}_{t}\left({a}_{i}\right)}},$$

where $0<\beta$ is a free parameter representing inverse temperature, i.e., the level of noise in decisions. This decision rule attributes higher probabilities to actions that are expected to yield high rewards.

Unlike our LSTM models that operate on sequences of four actions-rewards, the q-learning model is affected by the aggregation of the entire history of actions and rewards until the time point. This aggregation is shaped by the learning rate, such that the effect of previous actions and outcomes exponentially decays. However, unlike the LSTM model that keeps track of the ordering, in q-learning the specific order of actions and rewards is lost in the aggregation process.

The model was optimised for all participants’ entire sequence of actions and rewards, by maximising the log likelihood of actions (aggregated log probabilities of observed actions) using Eqs. (2) and (3), with SciPy's optimisation package (using L-BFGS-B). The optimisation process yielded an estimate of parameters $\alpha$ and $\beta$ for the entire population. This process also allowed recovery of trial-by trial Q and p(action) values for each of the four actions.

To calculate the accuracy of the reward-oriented model we chose the highest q-value in each time point as the model’s prediction, and compared it to the participant’s actual choice. The model’s accuracy was not calculated for trials where the participant did not make a choice.

For simulations of the model with new action-reward sequences we used the population estimated parameters, and initiated the Q values of all options with value of 50.

### Model comparison

To measure the similarity of the models’ performance over time (Fig. 3C), we compared the pattern of accuracy across participants for each trial, i.e., whether the models were accurate or inaccurate in predicting the same participants’ actions:

4 $${Similarity}_{t}= \frac{\sum_{i=1}^{M}{A}_{t}^{1,i}\oplus {A}_{t}^{2,i} }{M},$$

where ${A}_{t}^{k,i}$ is the accuracy of the model’s prediction at time $t$ for participant $i=1,..,M$ for model $k=\text{1,2}$ and $\oplus$ is XOR operation.

### Experimental simulations

To identify how reward-oriented and reward-oblivious predictions differ, and how they can be used to characterise the operation of the exploratory DNN model, we used experimental simulation. We used the LSTM models trained on the participants’ data as described above to generate action predictions for the exploratory and reward-oblivious models. To simulate the reward-oriented model we used a q-learning algorithm with the group-level parameters estimated from the model-fitting procedure, with the Q values of all options initiated at the value of 50.

The experimental simulations included 3 types of action patterns: Constant (a–a–a–a), Alternating (a–b–a–b) and All-different (a–b–c–d). Three types of reward patterns were used: Constant (r1 = r2 = r3 = r4), Ascending reward (r1 < r2 < r3 < r4), and Descending reward. We varied the exact rewards in each reward type and their timing in the one-different patterns. We also varied the specific actions used in the action patterns. An example of such action-reward combination is in Table 1.

Models’ predicted probabilities of choices were obtained for each choice following the action-reward combinations (see Table 1 for example), and were scored for distance between the predicted probabilities, using a symmetric Kullback–Leibler (KL) distance:

5 $$SymKL\left({P}_{M1},{P}_{M2}\right)=0.5\cdot {\sum }_{a=1}^{4}{P}_{M1}\left(a\right)\cdot \text{log}\left(\frac{{P}_{M1}\left(a\right)}{{P}_{M2}\left(a\right)}\right)+0.5\cdot {\sum }_{a=1}^{4}{P}_{M2}\left(a\right)\cdot \text{log}\left(\frac{{P}_{M2}\left(a\right)}{{P}_{M1}\left(a\right)}\right).$$

Pairwise comparison of the models under the different action-reward patterns is presented in Fig. 4. A link to all the model’s simulations is available in the Supplementary Material.

## Supplementary Information

Supplementary Information 1.

Supplementary Information 2.

Supplementary Information 3.

Supplementary Information 4.

## Data Availability

The in-house code developed for analysis is available here: https://osf.io/4cwme/.
