# Supplementary material for: Using deep learning to predict human decisions and using cognitive models to explain deep learning models
Source: Sci Rep. 2022 Mar 18;12:4736. doi: 10.1038/s41598-022-08863-0 (PMC8933393; doi:10.1038/s41598-022-08863-0)
Supplement: Supplementary file 1 — Supplementary Information 1. [file 41598_2022_8863_MOESM1_ESM.docx]

Supplementary materials

Using Deep Learning to Predict Human Decisions and Using Cognitive Models to Explain Deep Learning Models

Authors:

Matan Fintz 1, Margarita Osadchy 1, Uri Hertz 2

1. Department of Computer Science, University of Haifa, Haifa, Israel
2. Department of Cognitive Sciences, University of Haifa, Haifa, Israel

**Supplementary Methods – Task**

We examined a dataset of human decision making in a four-armed bandit task collected by Bahrami and Navajas. The dataset is available online: [OSF | 4 Arm Bandit Task Dataset](https://osf.io/f3t2a/)

The experiment was carried out online, and included 965 participants playing 150 rounds of a four-armed bandit task. In this task participants had to choose one of four options in each trial, in order to obtain rewards (points in the game, no performance based monetary reward was given in this experiment). The amount of rewards associated with each option was initially set to a value between 0 and 98 points, and drifted over time (standard deviation σ = 2.8):

$$\epsilon\left( t \right)\sim N\left( 0,2.8 \right)$$

$$r\left( t+1 \right)=r\left( t \right)+\epsilon(t)$$

Three different patterns of rewards were generated and then fixed. These fixed patterns were used for all participants. This procedure followed the method used in Daw et al. 2006 [1].

**Supplementary Methods – Behavioural Measures**

Behavioural measures included: The percentage of participants who chose each option on each time point (0-1), The variance of the choices made by all participants. These were calculated for each payoff structure independently. These measures are presented in figures SF5-7, along with the variance in the rewards on each time point (low variance indicates that rewards from all options are similar).

**Supplementary Methods - LSTM**

Long Short Term Memory (LSTM) is a type of recurrent neural networks (RNN), which allows modelling temporal dynamic behaviour by incorporating feedback connections in their architecture [2]. LSTM networks have an internal mechanism called gates that can regulate the flow of information and yield improved performance compared to vanilla RNN. Each LSTM unit includes a **memory cell** $\boldsymbol{c}$ (Eq. 5) and three gates: an **input gate** $\boldsymbol{i}$ (Eq. 1), an **output gate** $\boldsymbol{o}$ (Eq. 2) and a **forget gate** $\boldsymbol{f}$ (Eq. 3), that operate on the flow of information arriving in the cell via input variable $x^{<t>}$and feedback variable $h^{<t-1>}$. The architecture of the LSTM unit is shown in Figure 6 and the equations for the forward pass are summarised in Eq. 4-6.

$i= \sigma\left( {W_{i}[h}^{<t-1>}, x^{<t>}]+ b_{i} \right)$ (1)

$o=\sigma\left( W_{0}\left[ h^{\left\langle t-1 \right\rangle}, x^{<t>} \right]+b_{o} \right)$ (2)

$f=\sigma\left( W_{f}\left[ h^{\left\langle t-1 \right\rangle},x^{\left\langle t \right\rangle} \right]+b_{f} \right)$ (3)

$\tilde{c}^{\left\langle t \right\rangle}=\tanh(W_{c}\left[ h^{<t-1>}, x^{<t>} \right]+ b_{c})$ (4)

$c^{<t>}=i ⨀ \tilde{c}^{\left\langle t \right\rangle}+f ⨀ \tilde{c}^{\left\langle t-1 \right\rangle}$ (5)

$h^{<t>}=o ⨀ \tanh c^{<t>}$ (6)

Supplementary Figures


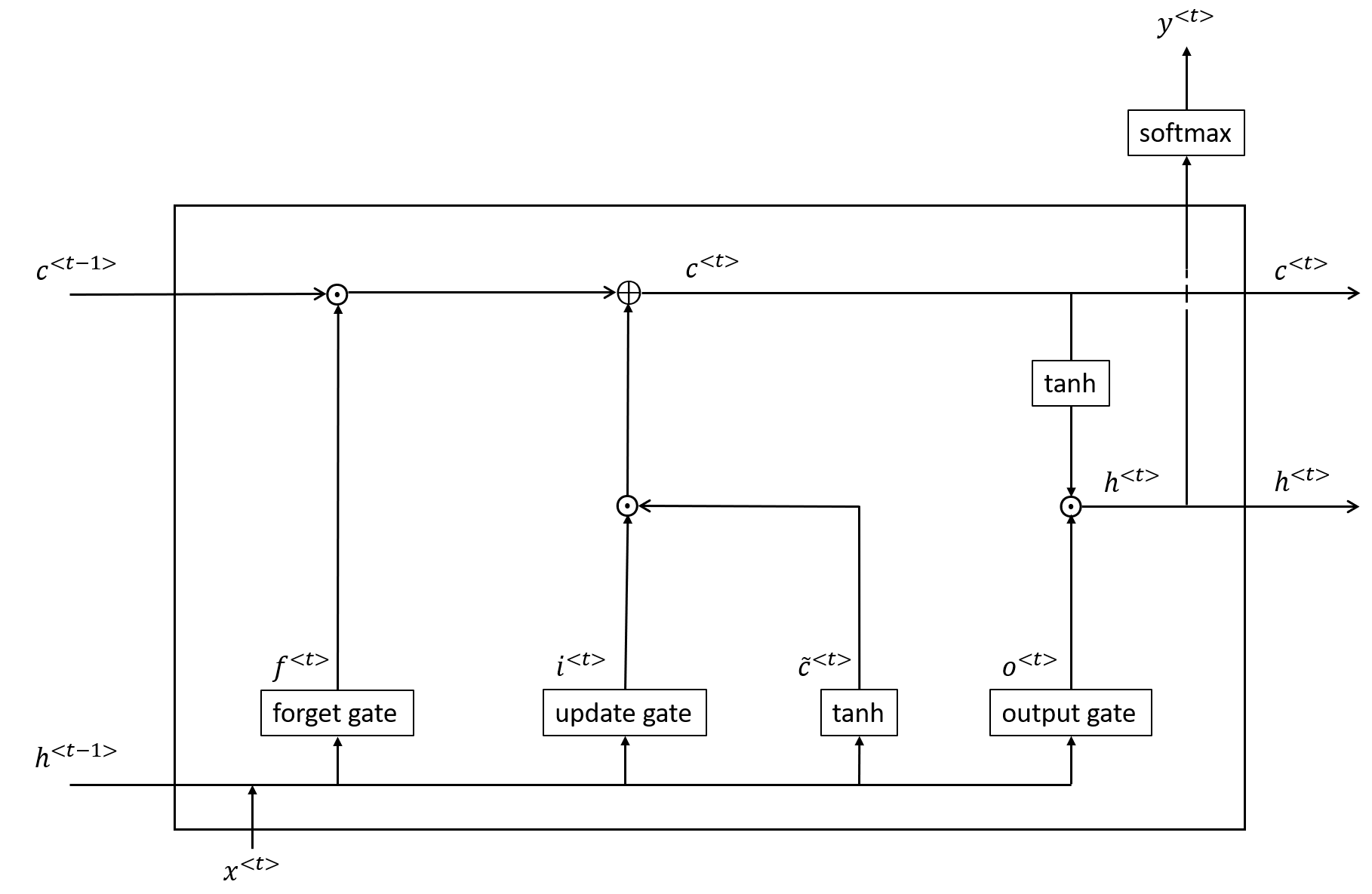


**SF1:** A LSTM cell. $\tilde{c}^{\left\langle t \right\rangle}$is a candidate for replacing the current cell state using previous feedback and current input [Eq. 4]. The new cell state $c^{\left\langle t \right\rangle}$ uses the forget and input gate (based on the previous step) along with the current candidate cell state to determine whether the cell state is to be updated or not. The next cell will get the final feedback$h^{<t>}$and if it is the last cell in the sequence, a 4-way softmax receives the feedback and makes a prediction for the current time t.

**
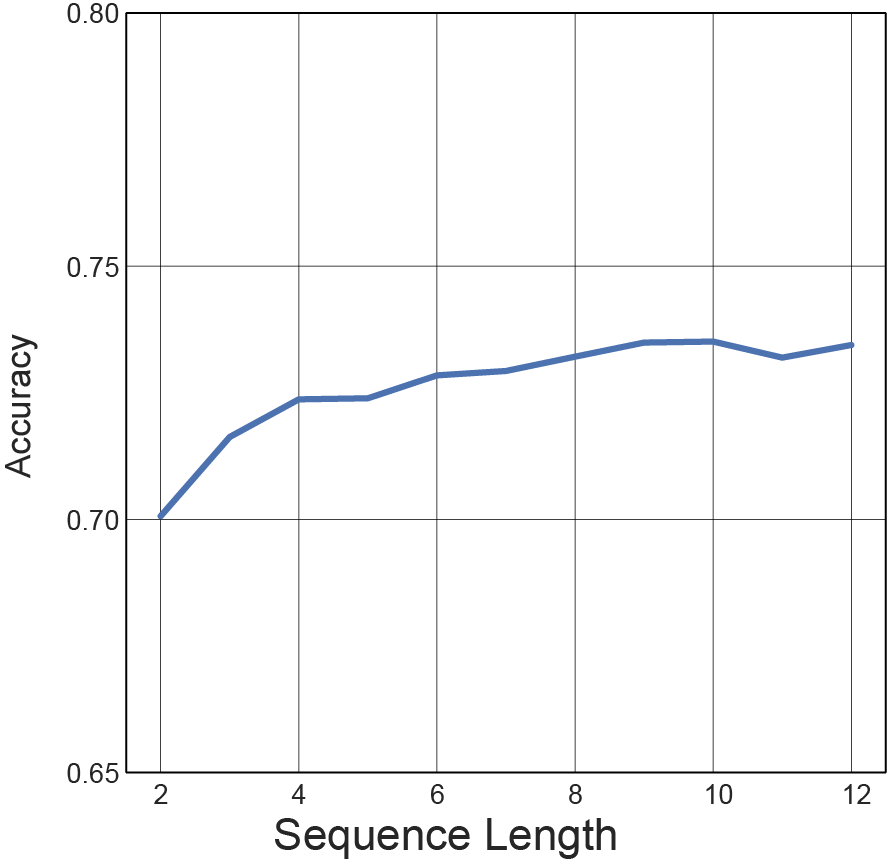
**

**SF2:** Accuracy of LSTM models with different sequence lengths. We trained LSTM models using different action-outcome sequence lengths, ranging from 2-12. The graph depicts the accuracy of each model in predicting participants’ actions. After evaluating the gain in accuracy made by the addition of each step, we chose 4-step sequences as a tradeoff between efficient use of the data and the memory needed for successful performance of the task.


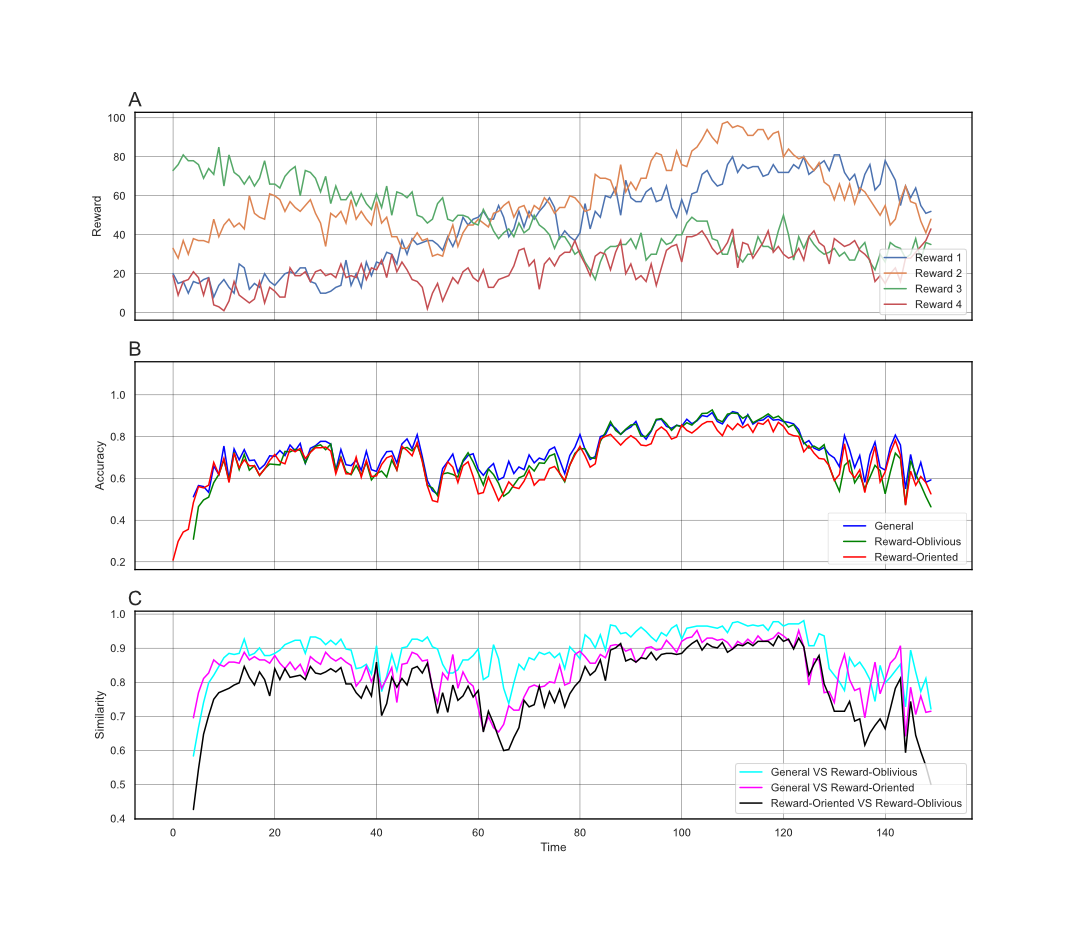


**SF3:** Analysis of models’ prediction over time – payoff structure 2. (A) Payoff structure indicates times when all options were similar and times when one option was distinctively better than in payoff #2. (B) Models’ prediction accuracy on trial-by-trial basis. (C) Measure of similarity in predictions between the two models over time.


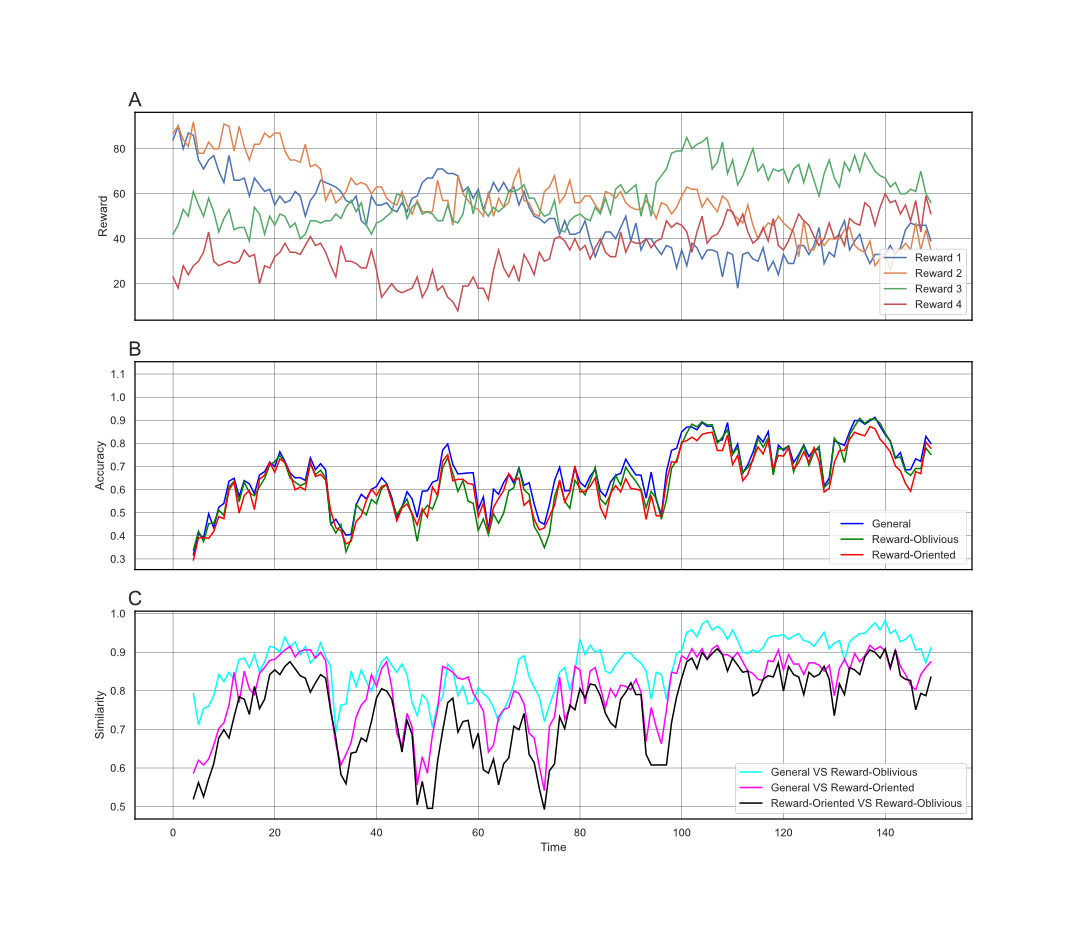


**SF4:** Analysis of models’ prediction over time – payoff structure 3. (A) Payoff structure indicates times when all options were similar and times when one option was distinctively better than in payoff #3. (B) Models’ prediction accuracy on trial-by-trial basis. (C) Measure of similarity in predictions between the two models over time.

**
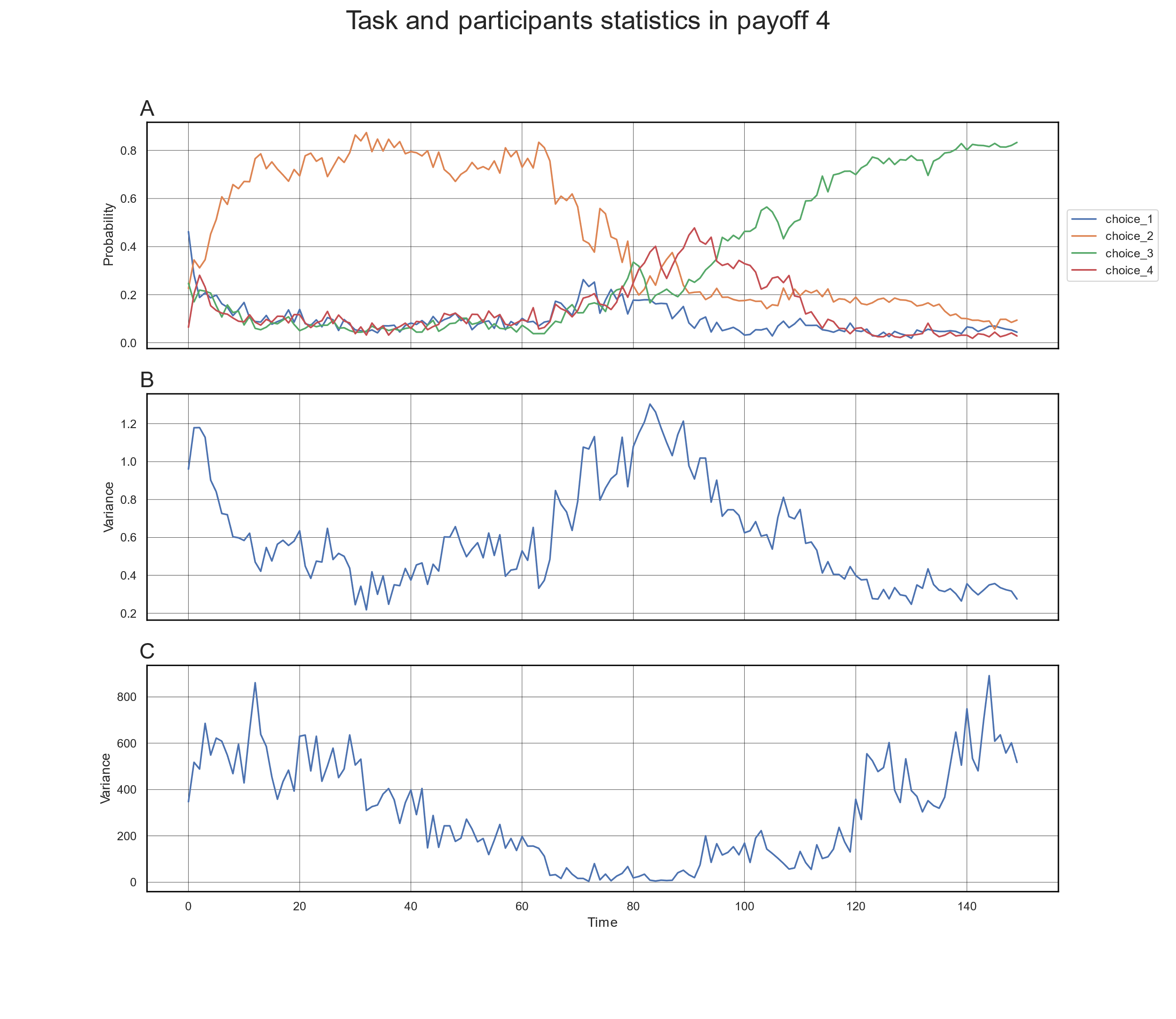
 SF5:** Participants’ performance in the original task with payoff structure #1. (A) Distribution of actual choices by participants over time (B) Variance of participants’ choices over time (C) Variance of possible rewards over time.

**
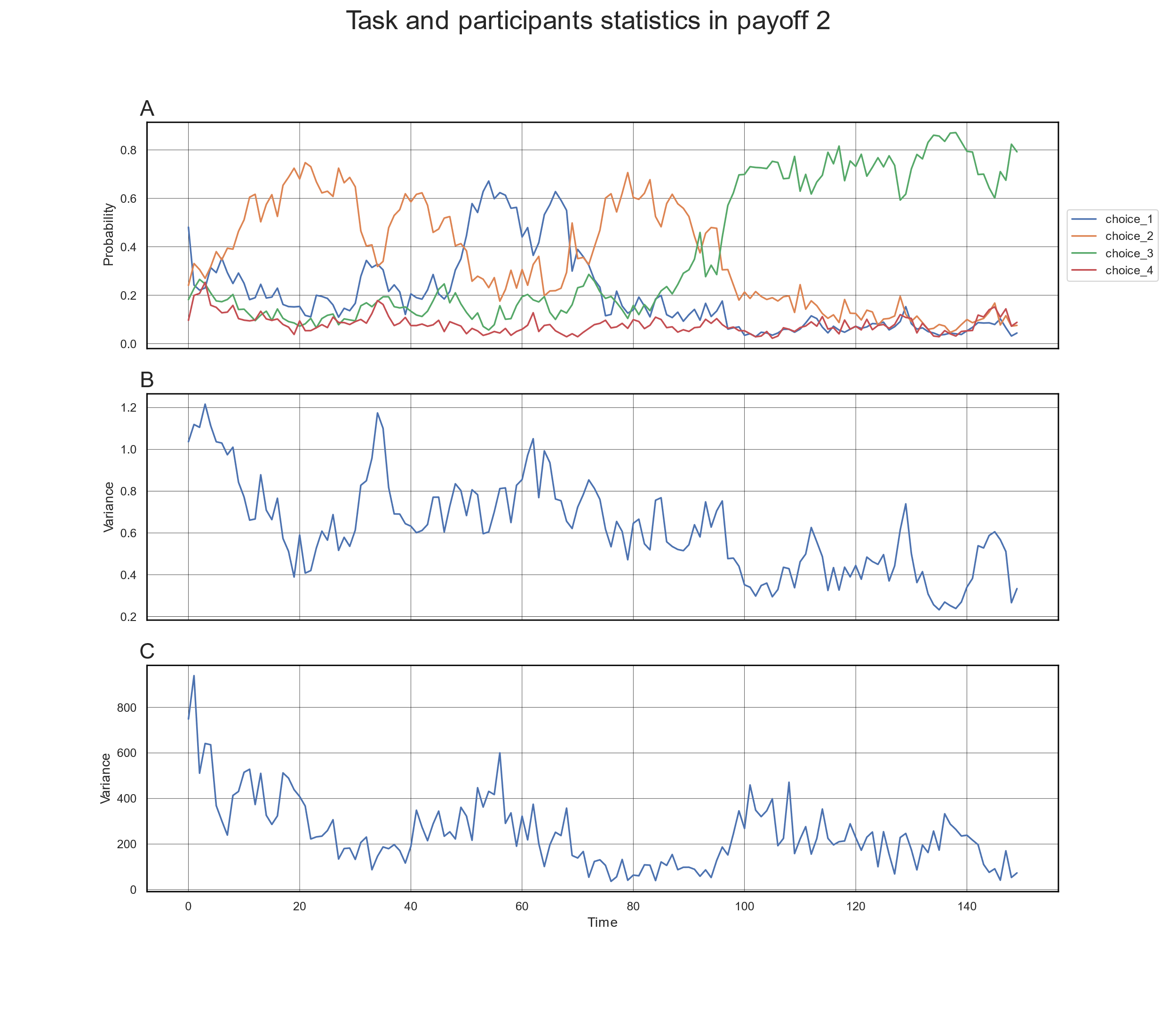
**

**SF6:** Participants’ performance in the original task with payoff structure #2. (A) Distribution of actual choices by participants over time (B) Variance of participants’ choices over time (C) Variance of possible rewards over time.

**
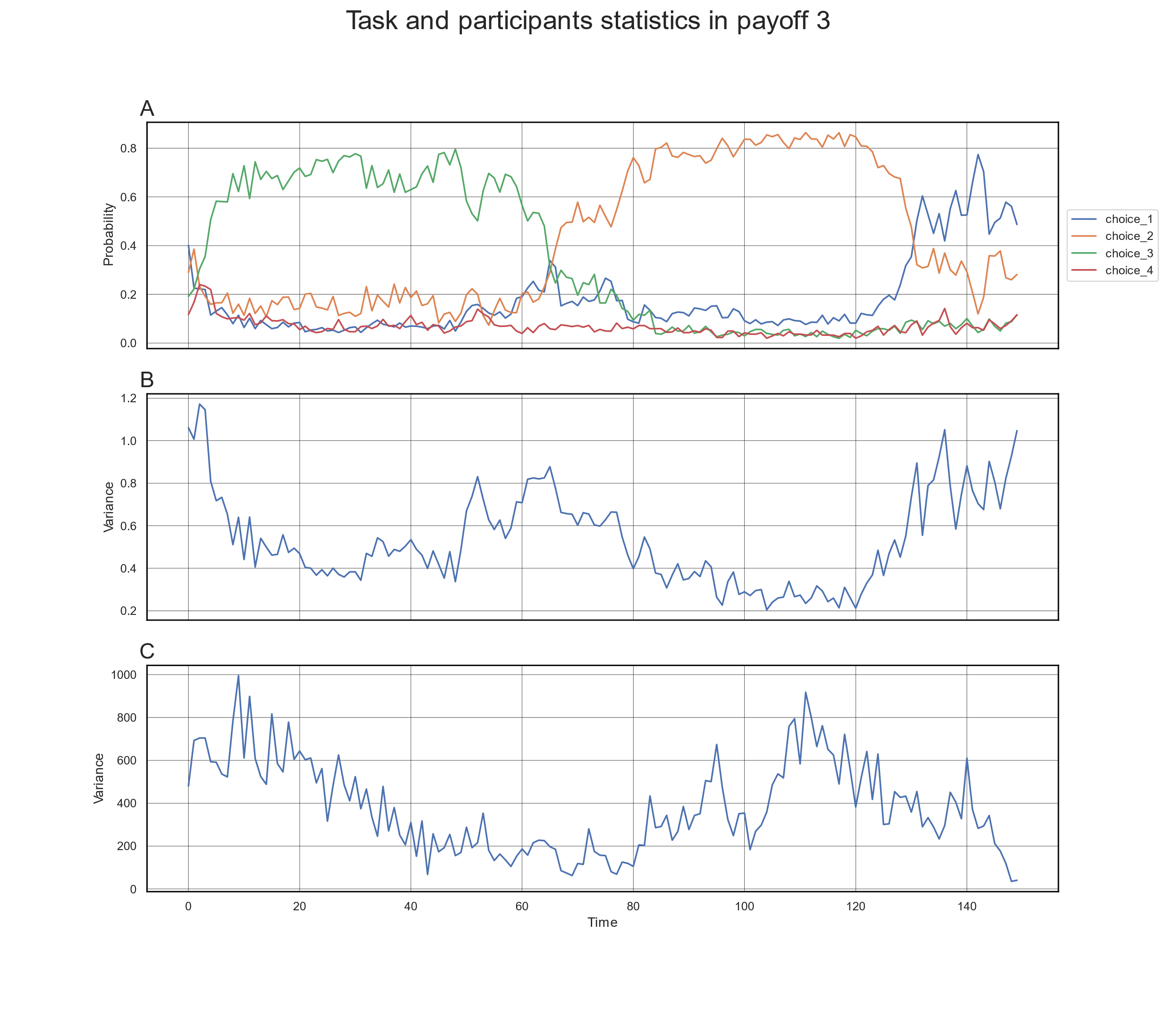
**

**SF7:** Participants’ performance in the original task with payoff structure #3. (A) Distribution of actual choices by participants over time (B) Variance of participants’ choices over time (C) Variance of possible rewards over time.


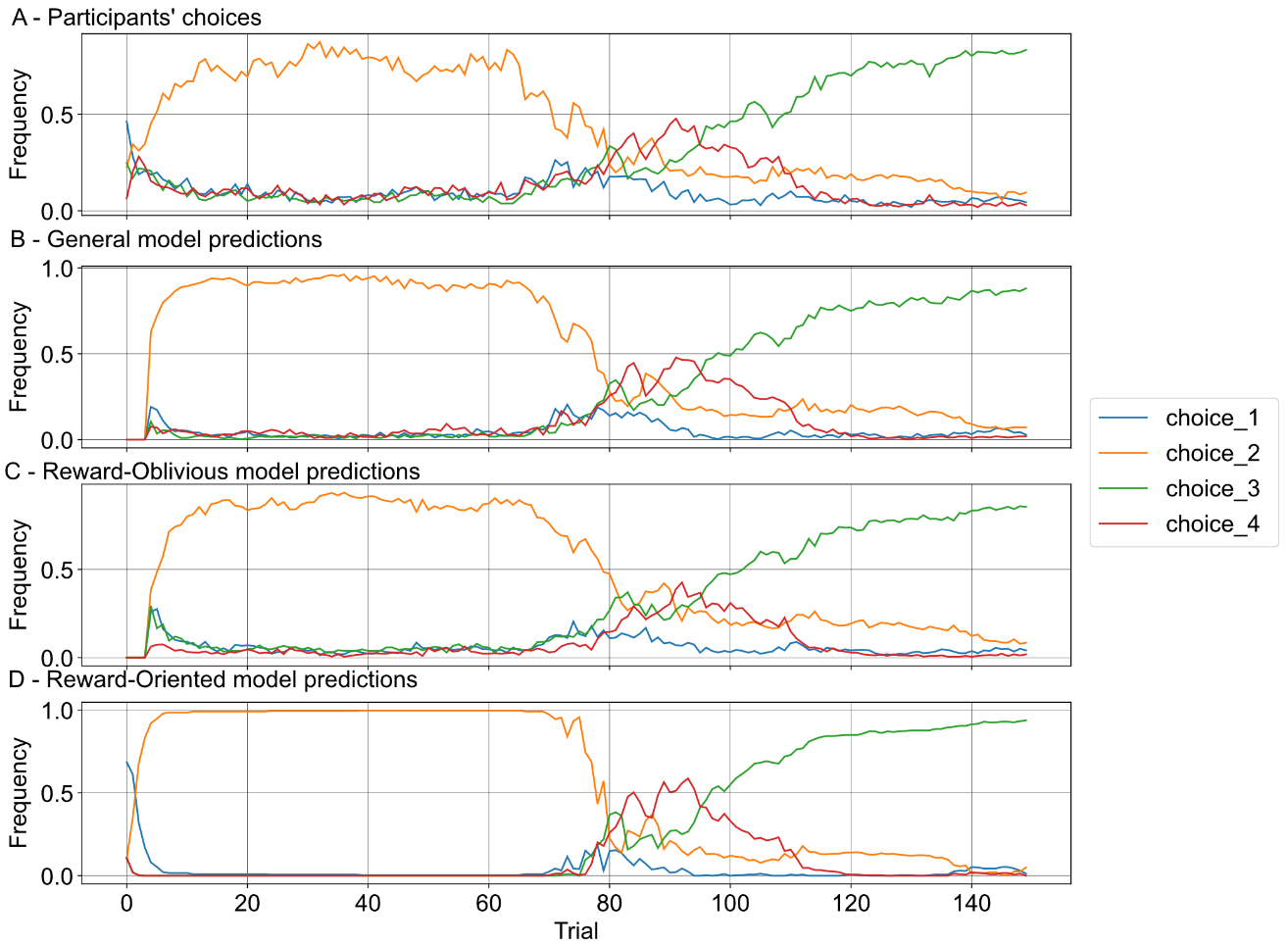


**SF8:** Models’ predictions and participants’ performance with payoff structure #1. The graphs present the average choices (predictions) in each time-point, indicating the portion of participants choosing (predicted to choose) each option at each time point.


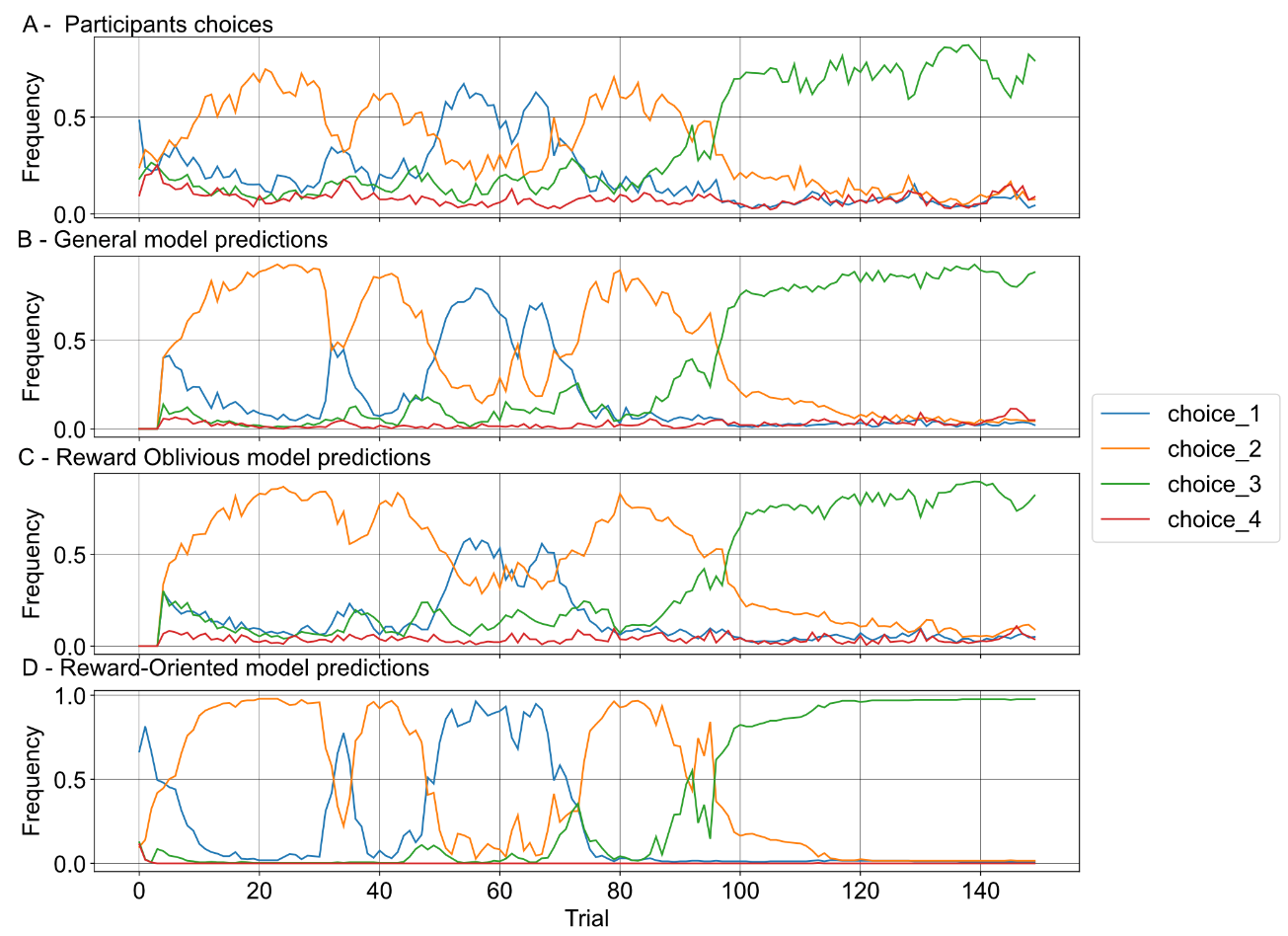


**SF9:** Models’ predictions and participants’ performance with payoff structure #2. The graphs present the average choices (predictions) in each time-point, indicating the portion of participants choosing (predicted to choose) each option at each time point.


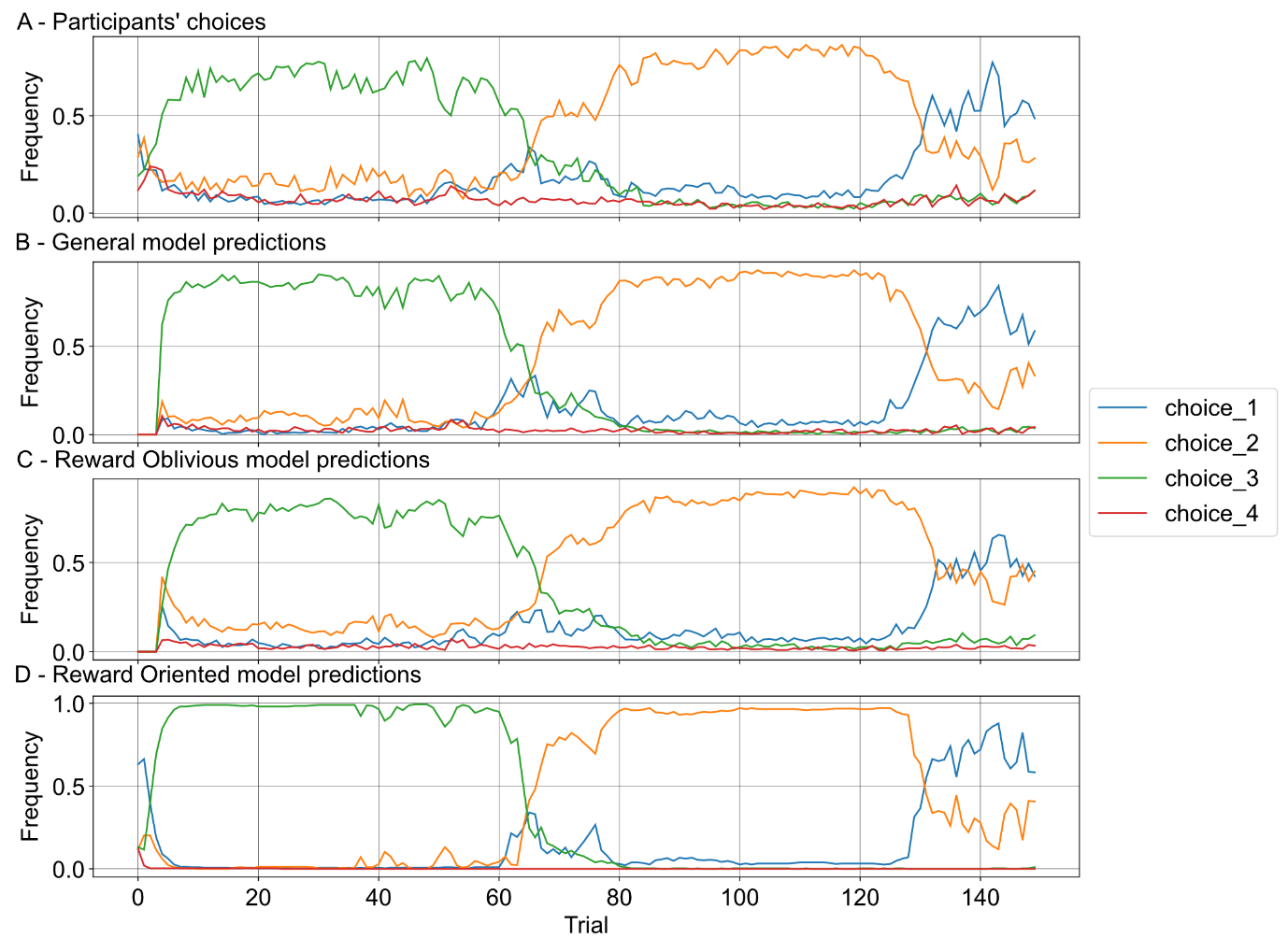


**SF10:** Models’ predictions and participants’ performance with payoff structure #3. The graphs present the average choices (predictions) in each time-point, indicating the portion of participants choosing (predicted to choose) each option at each time point.

**Supplementary tables (separate files)**

**ST1 – Experimental Simulations results for the reward-oblivious model. The softmax outcome for each action, in different combinations of outcome (x-axis) and action (y-axis) sequences.**

**The table is in file: no_reward_output_softmax_values_V5.1_paper_addition.csv**

**ST2 – Experimental Simulations results for the general DNN model. The softmax outcome for each action, in different combinations of outcome (x-axis) and action (y-axis) sequences.**

**The table is in file: general_output_softmax_values_V5.1_paper_addition.csv**

**ST3 – Experimental Simulations results for the reward-oriented model. The softmax outcome for each action, in different combinations of outcome (x-axis) and action (y-axis) sequences.**

**The table is in file: qlearning_output_softmax_values_V5.2_paper_addition.csv**
